# The *SbSOS1* gene from the extreme halophyte *Salicornia brachiata* enhances Na^+^ loading in xylem and confers salt tolerance in transgenic tobacco

**DOI:** 10.1186/1471-2229-12-188

**Published:** 2012-10-11

**Authors:** Narendra Singh Yadav, Pushp Sheel Shukla, Anupama Jha, Pradeep K Agarwal, Bhavanath Jha

**Affiliations:** 1 Discipline of Marine Biotechnology and Ecology, CSIR-Central Salt and Marine Chemicals Research Institute, (Council of Scientific and Industrial Research), G.B. Road, Bhavnagar, Gujarat, 364002, India

**Keywords:** *Salicornia brachiata*, *SbSOS1*, Transgenic plants, Salinity tolerance, Na^+^ loading

## Abstract

**Background:**

Soil salinity adversely affects plant growth and development and disturbs intracellular ion homeostasis resulting cellular toxicity. The *Salt Overly Sensitive 1* (*SOS1*) gene encodes a plasma membrane Na^+^/H^+^ antiporter that plays an important role in imparting salt stress tolerance to plants. Here, we report the cloning and characterisation of the *SbSOS1* gene from *Salicornia brachiata*, an extreme halophyte.

**Results:**

The *SbSOS1* gene is 3774 bp long and encodes a protein of 1159 amino acids. *SbSOS1* exhibited a greater level of constitutive expression in roots than in shoots and was further increased by salt stress. Overexpressing the *S. brachiata SbSOS1* gene in tobacco conferred high salt tolerance, promoted seed germination and increased root length, shoot length, leaf area, fresh weight, dry weight, relative water content (RWC), chlorophyll, K^+^/Na^+^ ratio, membrane stability index, soluble sugar, proline and amino acid content relative to wild type (WT) plants. Transgenic plants exhibited reductions in electrolyte leakage, reactive oxygen species (ROS) and MDA content in response to salt stress, which probably occurred because of reduced cytosolic Na^+^ content and oxidative damage. At higher salt stress, transgenic tobacco plants exhibited reduced Na^+^ content in root and leaf and higher concentrations in stem and xylem sap relative to WT, which suggests a role of SbSOS1 in Na^+^ loading to xylem from root and leaf tissues. Transgenic lines also showed increased K^+^ and Ca^2+^ content in root tissue compared to WT, which reflect that SbSOS1 indirectly affects the other transporters activity.

**Conclusions:**

Overexpression of *SbSOS1* in tobacco conferred a high degree of salt tolerance, enhanced plant growth and altered physiological and biochemical parameters in response to salt stress. In addition to Na^+^ efflux outside the plasma membrane, SbSOS1 also helps to maintain variable Na^+^ content in different organs and also affect the other transporters activity indirectly. These results broaden the role of *SbSOS1 in planta* and suggest that this gene could be used to develop salt-tolerant transgenic crops.

## Background

The productivity of over one-third of the arable land in the world is affected by the salinity of the soil [1]. More than 800 million ha of land worldwide are salt-affected [2]. High salinity adversely affects plant growth and development by disturbing intracellular ion homeostasis, which results in membrane dysfunction, attenuation of metabolic activity and secondary effects that inhibit growth and induce cell death [3]. Nearly all enzyme activity is reduced severely at NaCl concentrations above 0.3 M because of disruption of the electrostatic forces that maintain protein structure [4]. NaCl stress also significantly damages photosynthetic mechanisms through a combination of superoxide and H_2_O_2_-mediated oxidation [5]. Plants adapt to environmental stresses via a plethora of responses, including the activation of molecular networks that regulate stress perception, signal transduction and the expression of both stress-related genes and metabolites. Plants have stress-specific adaptive responses as well as responses which protect the plants from more than one environmental stress [6]. Numerous abiotic stress-related genes, as well as some transcription factors and regulatory sequences in plant promoters, have been characterised [7]. Plants employ three different strategies to prevent and adapt to high Na^+^ concentrations: 1) active Na^+^ efflux, 2) Na^+^ compartmentalisation in vacuoles, and 3) Na^+^ influx prevention [8,9]. Antiporters are an important group of genes that play a pivotal role in ion homeostasis in plants. Na^+^/H^+^ antiporters (NHX1 and SOS1) maintain the appropriate concentration of ions in the cytosol, thereby minimising cytotoxicity. NHX1 are located in tonoplasts and reduce cytosolic Na^+^ concentration by pumping in the vacuole [10], whereas SOS1 is localised at the plasma membrane and extrudes Na^+^ in apoplasts [11]. Both of these antiporters are driven by the proton motive force generated by the H^+^-ATPase [12].

The discovery of, and pioneer studies on, *sos* mutants in *Arabidopsis* uncovered a new pathway for ion homeostasis that promotes tolerance to salt stress. The *sos* mutants were specifically hypersensitive to high external concentrations of Na^+^ or Li^+^ and were unable to grow at low external K^+^ concentrations [13,14]. The SOS pathway consists of three proteins: SOS3, a calcium sensor protein [15]; SOS2, a serine/threonine protein kinase [16]; and SOS1 [17]. During salt stress, cellular Ca^2+^ levels are altered and CIPK and CBL interacting proteins are activated. Calcineurin B-like proteins (CBL) participate in salt stress-mediated signal transduction to control the influx and efflux of Na^+^[18]. The calcineurin B-like (regulatory) Ca^2+^ sensor *SOS3* (*Salt overly sensitive 3*) has been cloned from *Arabidopsis*[15]. SOS3 interacts with and activates the serine/threonine protein kinase SOS2 [16,19]. This interaction has been reported to recruit SOS2 to the plasma membrane where it interacts with SOS1 [20]. The *A. thaliana SOS1* gene was ectopically expressed for the first time in *Arabidopsis* and reduced the accumulation of Na^+^ in the presence of salt stress [21]. Similar results were obtained when *SOD2* and *NhaA,* which are plasma membrane Na^+^/H^+^ antiporters from *Schizosaccharomyces pombe* and *Escherichia coli*, respectively, were overexpressed in *Arabidopsis*[22] and rice [23]. Heterologous expression of different plant *SOS1* genes suppressed the Na^+^ sensitivity of the yeast mutant (AXT3K) (*Arabidopsis thaliana,*[11]; *Arabidopsis thaliana,*[24]; *Cymodocea nodosa,*[25]; *Oryza sativa,*[26]; and *Solanum lycopersicum,*[27]). Additionally, Wu et al. [28] and Garciadeblas et al. [25] showed that expression of *PeSOS1* and *CnSOS1* partially suppressed the salt-sensitive phenotypes of the EP432 bacterial strain (*nhaA,nhaB*), which lacks activity of the two Na^+^/H^+^ antiporters *EcNhaA* and *EcNhaB*.

Few studies have performed *in planta* overexpression of the *SOS1* gene from *Arabidopsis*[21,29], *Thellungiella*[30] and *Puccinellia tenuiflora*[31]. The study of the salt tolerance mechanisms of halophytic plants has emerged as an important area because these species are well-adapted to and can overcome soil salinity more efficiently than glycophytic plants [32]. Our lab studies an extreme halophyte, *Salicornia brachiata* Roxb. (*Amaranthaceae*), in an effort to identify and characterise novel genes that enable salt tolerance. *S. brachiata*, a leafless succulent annual halophyte, commonly grows in the Gujarat coastal marshes in India. *Salicornia* can grow in a wide range of salt concentrations (0.1–2.0 M) and can accumulate quantities of salt as high as 40% of its dry weight. This unique characteristic provides an advantage for the study of salt tolerance mechanisms. *Salicornia* accumulates salt in the pith region, which reflects the fact that antiporter genes are necessary to maintain homeostasis in extreme salinity. This report describes the isolation of the *SbSOS1* gene from *S. brachiata* and overexpression in tobacco plant for functional validation.

## Methods

### Plant growth and salt treatments

*Salicornia brachiata* Roxb. seeds were collected from dried plants obtained from the coastal area near Bhavnagar, Gujarat, India. The seeds were germinated in plastic pots containing garden soil and the plants were grown in natural conditions. At one month, the seedlings were carefully uprooted and transferred to hydroponic conditions (¼ major and minor MS stock) in a culture room with a dark/light cycle of 8/16 h at 25°C for one month. The plants were treated with various concentrations of NaCl (0.10, 0.25, 0.5, 1.0, 1.5, and 2.0 M) for 48 h. Upon completion of the treatments, shoot and root tissues were separately collected, frozen in liquid nitrogen and stored at −80°C.

### Cloning of *SbSOS1* gene

Degenerate primers were designed from conserved regions of the *SOS1* gene. To identify conserved regions of the *SOS1* gene for PCR amplification, *SOS1* cDNA sequences from *Arabidopsis* (NM126259), *Chenopodium quinoa* (NM126259), *Suaeda japonica* (AJ717346), *Mesembryanthemum crystallinum* (EF207776) *Solanum lycopersicum* (AJ717346), *Oryza sativa* (AY785147), and *Triticum aestivum* (AY326952) were retrieved from NCBI and aligned with the ClustalW program. On the basis of sequence conservation across species, five pairs corresponding degenerate primers were designed. Of these primers, DF2-DR2 (5^′^-GCTTGTCGTCACTTTCTTCG-3^′^ and 5^′^-CM(A+C)CCAAATGCH(A+T+C)TCTAATGC-3’) and DF5-DR4 (5^′^-TGCTTACTGGGYGATGCT-3^′^ and 5^′^-AGYCCCAAAGTACTTCCATG-3^′^) primer sets produced fragments of the desired size.

RNA was extracted from the shoots of *S. brachiata* using the GITC method. Total RNA (5 μg) was used in RT-PCR reactions and cDNA was synthesised with a Superscript RT II first strand cDNA synthesis kit (Invitrogen, San Diego, CA, USA). The *SOS1* gene was amplified in the presence of 10 pmol of the degenerate primers, 2.5 U Taq and 1 μl cDNA according to the following sequence: initial denaturation at 95°C for 2 min, 35 cycles of 94°C for 1 min, 55°C for 1 min, 72°C for 2 min, and an additional 10 min polymerisation step at 72°C. The amplified fragments were purified from agarose gels and cloned into the pGEM-T Easy vector system II (Promega, Madison, Wisconsin) and transformed into DH5α *Escherichia coli* cells. After sequencing these RT-PCR fragments (1300 bp and 750 bp), four additional primers were designed to amplify the full-length cDNA via rapid amplification of cDNA ends (RACE).

The 5^′^-RACE reaction was performed according to the manufacturer’s protocol (Invitrogen, San Diego, CA, USA). The first strand of cDNA was synthesised with a gene-specific primer GSP R1 (5^′^-AGAGTCAAGGGGGTTTCAATTC-3^′^) and Superscript RT II. The mRNA was removed with RNase H, and a homopolymeric tail was added to the 3^′^-end of the cDNA. PCR amplification was performed using a nested, gene-specific primer GSP R2 (5^′^-CTACAGCTACAGGATCAGTTGCAC-3^′^) and a dG-anchor primer AAP (5^′^-GGCCACGCGTCGACTAGTAC(G)_16_ -3^′^).

To perform 3^′^-RACE reactions, the first strand of cDNA was synthesized at the poly (A) mRNA tail with PK1 oligo dT adapter primer (5^′^-CCAGTGAGCAGAGTGACGAGGACTCGAGCTCAAGC(T)_17_-3^′^). Following the first strand of cDNA synthesis, PCR was performed with a gene-specific primer, GSP F1 (5^′^-AGTAGTAAAGACCAGGCAAGCAAC-3^′^), and an adaptor primer, PK2 (5^′^-CCAGTGAGCAGAGTGACG-3^′^). The 5^′^- and 3^′^-RACE products were cloned into a pGEM-T Easy vector. The cloned products of the original PCR and the 5^′^-and 3^′^-RACE reactions were sequenced, and contiguous sequences were assembled to obtain the full-length *SbSOS1* gene. After determining the open reading frame, the full length *SOS1* cDNA was amplified with AccuPrime Pfx DNA polymerase (Invitrogen), using SbSOS F (5^′^-ATCGGGGTACCATATGGCAGCATCTCGAATTGA-3^′^) and SbSOS R (5^′^-ATTCCCCCGGGTCAAGGAGCTTGGCGGAA-3^′^) primers. The amplification product was then cloned into a pJET1.2/blunt cloning vector (MBI Fermentas) and sequenced by primer walking (Macrogen Inc., Seoul, South Korea).

### *In silico* analysis

The NCBI database was searched for nucleotide and protein sequences. TMpred online software was used to predict transmembrane domains, and DNAMAN was used to align sequences. Conserved domains in SbSOS1 were identified via the BLASTp program (http://www.ncbi.nlm.nih.gov). ExPASy tools (http://www.expasy.ch/tools/) was used to predict secondary structures. The relationship of SbSOS1 with SOS1 from other plant species was inferred by constructing phylogenetic tree using MEGA Ver. 4.0. The robustness in topology of the phylogenetic tree was assessed based on bootstrap value.

### Quantitative real time PCR

Total RNA was isolated from control and NaCl-treated plant samples using GITC buffer and quantified with a Nanodrop spectrophotometer (USA). The cDNA was prepared with 5 μg of total RNA with a Superscript RT II first-strand cDNA synthesis kit (Invitrogen, San Diego, CA, USA). Real Time qPCR was performed in a Bio-Rad IQ5 detection system (Bio-Rad, U.S.A.) with 1x SYBR Green (Sigma, USA). The PCR reactions were carried out in 1x PCR buffer supplemented with 200 μM dNTPs, 1.25 U Taq DNA polymerase and 5 pmol of each gene-specific primer. The PCR reactions were performed under the following conditions: initial denaturation at 95°C for 5 min, 40 cycles at 95°C for 10 sec, 60°C for 30 sec and 72°C for 30 sec. At the end of the PCR cycles, the products were subjected to melt curve analysis to verify the specificity of PCR amplification. The amplified products were electrophoresed through 1.2% agarose gel to verify that they matched the predicted size. Three independent experiments were performed with three replicates each. Fold changes were calculated using the CT method [33], and CT values for individual variants were compared to those of a reference control (*β-tubulin*). The *SbSOS1*-specific primer pair RT F (5^′^-GGAAGGTTTGGGGATGGTAT-3^′^) and RT R (5^′^-GTCCAGCAAGCAAAACCATT-3^′^) were utilised for expression study of *SbSOS1,* whereas QBT F (5^′^-GGAGTCACCGAG GCAGAG-3^′^) and QBT R (5^′^-ATCACATATCAGAAACCACAA-3^′^) primers were used for an internal control.

### Construction of plant transformation vector and tobacco transformation

To perform plant transformation, *SbSOS1* cDNA was PCR-amplified with AccuPrime Pfx DNA polymerase (Invitrogen) in conjunction with UA F (5^′^-TAACAGGGCCCATGGCAGCATCTCGAATTGA-3^′^) and UK R (5^′^-ATCGGGGTACCTCAAGGAGCTTGGCGGAA-3^′^) primers, which contained *Apa*I and *Kpn*I sites, respectively. The digested *SbSOS1* gene was cloned as an *Apa*I/ *Kpn*I fragment into the pRT 100 vector [34]. Thereafter, the entire cassette containing the CaMV 35S constitutive promoter and both the *SbSOS1* gene and terminator were cloned into the pCAMBIA2301 vector at the *Pst*I site. The resulting vector was mobilised into *Agrobacterium tumefaciens* (LBA 4404) and used to transform tobacco (*Nicotiana tabacum* cv. Xanthi) plants according to a standard protocol [35]. Putative transgenic plants regenerated directly from leaf edges in the presence of kanamycin (50 mg/l) were transferred in jam bottles that contained MS basal medium [36] supplemented with kanamycin (100 mg/l). The transgenic lines were screened via GUS assay, PCR amplification and semiquantitative PCR analysis. To obtain transgenic T_1_ seedlings, seeds from the parental plants (T_0_ seeds) were germinated on kanamycin-supplemented MS medium.

### Semiquantitative PCR analysis

Total RNA was isolated from WT and transgenic plant samples using GITC buffer and was quantified with a Nanodrop spectrophotometer (USA). The cDNA was prepared using 5 μg of total RNA with a Superscript RT III first-strand cDNA synthesis kit (Invitrogen, San Diego, CA). The synthesised cDNA (1 μl, diluted 1:5) was used as a template, and actin was used as an internal control for RT-PCR analysis. The *SbSOS1*-specific primer pair, RT F (5^′^-GGAAGGTTTGGGGATGGTAT-3^′^) and RT R (5^′^-GTCCAGCAAGCAAACCATT-3^′^), was utilised for expression study of the *SbSOS1*, whereas QACT F (5^′^-CGTTTGGATCTTGCTGGTCGT-3^′^) and QACT R (5^′^- CAGCAATGCCAGGGAACATAG −3^′^) primers were used for actin. The PCR reactions were carried out in 1x PCR buffer supplemented with 200 μM dNTPs, 1.25 U Taq DNA polymerase and 5 pmol of each of the gene-specific primers according to the following conditions: an initial denaturation at 95°C for 5 min, 25 cycles at 94°C for 30 sec, 60°C for 30 sec and 72°C for 30 sec, followed by a final extension step at 72°C for 7 min. RT-PCR experiments were repeated three times, and the amplification products were analysed via agarose gel electrophoresis.

### Histochemical GUS staining

GUS activity was visualised in leaf tissue with 1 mM 4-methyl-umbelliferyl-b-D-glucuronide as was described by Jefferson [37]. Leaves from control and transgenic plants were cut to 1 cm^2^ in size and rinsed in 50 mM phosphate buffer (pH 7.0). Leaf sections were then incubated with 1 mM 4-methyl-umbelliferyl-b-D-glucuronide prepared in 50 mM phosphate buffer, vacuum infiltrated for 10 min and then incubated overnight at 37°C in the dark. The tissues were then rinsed with 80% ethanol for 4 h to remove chlorophyll.

### Extraction of genomic DNA and PCR analysis

Genomic DNA was isolated from different T_0_ lines via the CTAB (N-cetyl-N,N,N-trimethylammonium bromide) method. To verify the presence of the transgene, PCR was conducted with gene-specific primers for real-time PCR (RTF and RTR) and GUS-specific primers (gusA F 5^′^-GATCGCGAAAACTGTGGAAT-3^′^ and gusA R 5^′^-TGAGCGTCGCAGAACATTAC-3^′^).

### Leaf disc assay and estimation of chlorophyll

Leaf discs from WT and two T_0_ transgenic lines (L1 and L7) were used in the salt tolerance assay. The leaf discs were floated on ¼ MS (control) and different concentrations of NaCl for 15 days. The leaf discs were then homogenised thoroughly in 80% acetone and centrifuged at 3,000 ×*g* for 2–3 min. The O.D. of each supernatant was recorded at 663 and 645 nm, and the chlorophyll content was calculated per gram of fresh tissue weight [38]. The treatments were conducted under continuous exposure to white light at 25 ± 2°C.

### Evaluation of transgenic plants exposed to salt stress

To analyse the salt stress tolerance of *SbSOS1*-overexpressing tobacco plants, the seeds from T_0_ transgenic plants were germinated in MS medium supplemented with 0, 50, 100, 150, 200, or 300 mM NaCl in culture room conditions. The percentage of seed germination was scored 15 days after seed inoculation. T_1_ seedlings were also analysed for different growth parameters under salt stress. At eight days, WT and kanamycin-positive T_1_ seedlings (50 mg/l) were transferred to MS medium supplemented with 0, 100 or 200 mM NaCl in petridish. Shoot length, root length, leaf surface area, fresh weight, dry weight and relative water content (RWC) of the seedlings were measured after 30 days of growth. At one month, WT and kanamycin-positive T_1_ seedlings were transferred into beakers containing 1/2 MS hydroponic culture and were maintained therein for 20 days prior to receiving stress treatments of 0, 100 or 200 mM NaCl. Leaf, shoot and root tissues were collected 45 days post-treatment and subjected to physiological and biochemical analyses. For ROS analysis 15-day-old WT and kanamycin-positive T_1_ seedlings were transferred to MS medium supplemented with 0, 100, 200 and 300 mM NaCl in culture jars and maintained for 60 days.

### Membrane stability index (MSI)

MSI was determined as described by Hayat et al. [39]. In brief, MSI was estimated by taking leaf samples in 10 ml of double-distilled water in two sets. One set was heated at 40°C for 30 min in a water bath and measured for electrical conductivity (C_1_). The second set was boiled at 100°C for 10 min prior to having its conductivity (C_2_) measured. MSI was calculated according to the formula [1] as described by Sairam [40]:

(1) $$\text{MSI}=\left(1-C_{1}/C_{2}\right)$$

### Electrolyte leakage

Electrolyte leakage was measured as described by Lutts et al. [41]. Young leaves (3^rd^ leaf from the top) of similar size were collected from four plants for each treatment and washed thoroughly with deionised water to remove surface-adhered electrolytes. The samples were placed in closed vials containing 10 ml of deionised water, incubated at 25°C on a rotary shaker for 24 h. Subsequently, the electrical conductivity of the solution (*L*_t_) was determined using a SevenEasy conductivity meter (Mettler Toledo AG 8603, Switzerland). The samples were then autoclaved at 120°C for 20 min and cooled at 25°C before determining the final electrical conductivity (*L*_0_). Electrolyte leakage was defined as follows:

(2) $$\text{Electrolyte leakage}\;\left(\%\right)\;=\left(L_{t}/L_{0}\right)\times 100$$

### Lipid peroxidation

Lipid peroxidation was estimated by determining the concentration of malondialdehyde (MDA) produced by the thiobarbituric acid (TBA) reaction; this experiment was performed following the method described by Hodges et al. [42]. Leaf material (0.5 g) was homogenised in 15 ml of an 80% acetone solution. In one set, 1 ml of extract was added to 1 ml 0.5% (w/v) TBA in 20% (w/v) TCA. In another set, TBA was excluded. The mixture was incubated at 90°C for 30 min and then cooled at room temperature. The samples were centrifuged at 4,000 rpm for 3 min and the absorbance of the supernatants at 440 nm, 532 nm and 600 nm was determined. The MDA concentration was derived according to the following equations:

1)

(3) $$\begin{array}{cc} \;A= & \left[\left({\text{Abs}}_{532+\text{TBA}}-{\text{Abs}}_{600+\text{TBA}}\right)\right)\\ & -\left(\left({\text{Abs}}_{532-\text{TBA}}-{\text{Abs}}_{600-\text{TBA}}\right)\right] \end{array}$$

2)

(4) $$B=\left[\left({\text{Abs}}_{440+\text{TBA}}-{\text{Abs}}_{600-\text{TBA}}\right)0.0571\right]$$

3)

(5) $$\begin{array}{c} \text{MDA equivalents}\left({\text{nmol gm}}^{-1}\right)\\ \;=\left(A-B/15,700\right)10^{6} \end{array}$$

### *In vivo* localisation and quantification of O_2_^-^ and H_2_O_2_ content in the leaves of T_1_ plants

*In vivo* detection of O_2_^−^ and H_2_O_2_ was accomplished by histochemical staining with nitro blue tetrazolium (NBT) and 3, 3^′^- diaminobenzidine (DAB) as described by Shi et al. [43]. The presence of O_2_^−^ and H_2_O_2_ in transgenic and WT leaves exposed to salt stress was detected by immersing the leaf samples in room temperature solutions of NBT (1 mg ml^−1^) and DAB (1 mg ml^−1^, pH 3.8) in 10 mM phosphate buffer (pH 7.8). To detect O_2_^−^, immersed leaves were illuminated for 12 h until blue spots appeared, which are indicative of formazan precipitates. To determine the localisation of H_2_O_2_, immersed leaves were incubated in the light at room temperature for 24 h until brown spots became visible; these spots occur due to the reaction of DAB with H_2_O_2_. Following incubation, the leaf chlorophyll was bleached in absolute ethanol to enable visualisation of the blue and brown spots.

O_2_^-^ content was determined according to Liu et al. [44]. Leaf tissue was extracted in 10 ml of 65 mM potassium phosphate buffer (pH 7.8) and centrifuged at 5,000 ×g for 10 min. The reaction mixture containing 0.9 ml of 65 mM phosphate buffer (pH 7.8), 0.1 ml of 10 mM hydroxylamine hydrochloride, and 1 ml of the extract was incubated at 25°C for 20 min. Then 17 mM sulfanilamide and 7 mM α-naphthylamine were added, further incubated at 25°C for 20 min and the absorbance was read at 530 nm. A standard curve (10–200 nmol) was prepared with NaNO_2_, to calculate the production rate of O_2_^-^.

The H_2_O_2_ content in leaf samples was measured as described by Mukherjee and Choudhuri [45]. Leaf tissue was extracted with cold acetone to determine the H_2_O_2_ levels. 2 ml of the extract was mixed with 0.5 ml of 0.1% titanium dioxide in 20% (v:v) H_2_SO_4_ and the mixture was then centrifuged at 6,000 x*g* for 15 min. The intensity of yellow colour of the supernatant was measured at 415 nm and the concentration of H_2_O_2_ was calculated against the standard curve.

### Measurement of proline, soluble sugar and amino acid content

Free proline content in the leaves was determined using acid ninhydrin as previously described [46] with minor modifications. Plant tissue (100 mg) was homogenised in 1.2 ml 3% aqueous sulphosalicylic acid and centrifuged at 13000 rpm for 10 min. After centrifugation, 500 μl of supernatant was diluted to 1 ml with distilled water, reacted with 1 ml of 2% ninhydrin in acetone and incubated at 90°C for 1 h. The samples were cooled on ice and 2 ml of toluene was added and vortexed for 2 min. The upper phase was aliquoted to determine absorbance at 520 nm in a T80+ UV–vis spectrophotometer (PG Instruments Ltd., U.K.). Proline content was calculated by comparing the value against a standard curve derived from known concentrations of L-proline (Sigma Aldrich, USA) and expressed as μg/mg of fresh weight. Total soluble sugars were analysed by treating 0.1 ml of the alcoholic extract with 3 ml of freshly prepared anthrone reagent (150 mg anthrone in 100 ml 72% (v/v) H_2_SO_4_). This mixture was placed in a boiling water bath for 10 min as described by Irigoyen et al. [47]. After the mixture was cooled, absorbance at 620 nm was measured with a T80+ UV–vis spectrophotometer. A calibration curve was constructed with glucose in the range 20–400 μg/ml (Sigma Aldrich, USA). Total amino acid content was determined as described by Shukla et al. [48]. Specifically, 1 ml of plant extract was treated with 1 ml of 0.2 M citrate buffer (pH 5.0), 1 ml of 80% ethanol and 1 ml ninhydrin (1%), followed by incubation at 95°C for 15 min. The samples were cooled and the absorbance at 570 nm was measured [49] with a T80+ UV–vis spectrophotometer.

### Na^+^, K^+^ and Ca^2+^ ion content analysis

Ion content was determined via the method described by Shukla et al. [48]. Plant tissue (0.2 g) was digested in 4 ml of a solution of perchloric and nitric acids (3:1). The solution was dried on a hot plate at 90°C and reconstituted to 25 ml with deionised water before filtration through a 0.2 μm microfiber filter. Xylem sap was collected according to the method described by Olias et al. [27]. The ion content of digested plant tissues (root, stem and leaf) and xylem sap was measured with an inductively coupled plasma optical emission spectrometer (Optima 2000DV, Perkin Elmer, Germany).

### Statistical analyses

Each experiment was performed three times and data from 10 plants were recorded. One-way ANOVA with replicates was performed in Microsoft Excel. C.D. values were calculated at *P* = 0.05 to determine the significance of difference between the means of WT and transgenic plants of each treatment group. Mean values that were significantly different within treatment from each other are indicated by different letters. The SD was calculated to show the variation in the replicates.

## Results

### *SbSOS1* encodes a putative Na^+^/H^+^ antiporter

**Figure 1:**
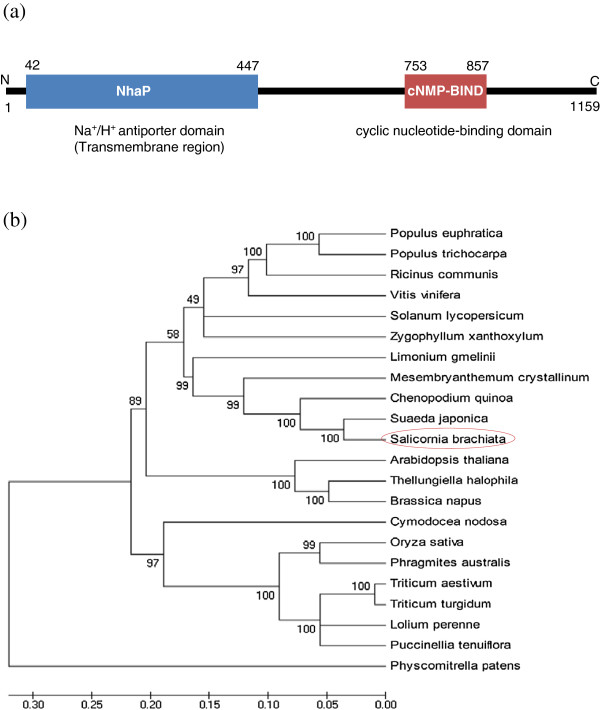
(**a**) **Schematic diagram showing the transmembrane domains and cyclic nucleotide**-**binding domain.** N and C indicates N-terminal and C-terminal end of the SbSOS1, respectively. (**b**) The phylogenetic relationship of SbSOS1 with SOS1 from other plant species. The phylogenetic tree was constructed using MEGA ver. 4.0 and the bootstrap values were calculated from 100 replicates and are shown next to branches. The higher bootstrap value signifies resilience in the phylogenetic position of the protein. The scale bar indicates substitutions per site. The protein sequences used for construction of the phylogenetic tree are as follows: *Suaeda japonica* (BAE95196.1), *Chenopodium quinoa* (ACN66494.1), *Mesembryanthemum crystallinum* (ABN04858.1), *Limonium gmelinii* (ACF05808.1), *Ricinus communis* (XP_002521897.1), *Populus trichocarpa* (XP_002315837.1), *Vitis vinifera* (ACY03274.1), *Populus euphratica* (ABF60872.1), *Zygophyllum xanthoxylum* (ACZ57357.1), *Solanum lycopersicum* (BAL04564.1), *Cymodocea nodosa* (CAD20320.1), *Phragmites australis* (BAF41924.1), *Oryza sativa Japonica* (AAW33875.1), *Triticum turgidum* (ACB47885.1), *Puccinellia tenuiflora* (BAK23260.1), *Triticum aestivum* (CAX83738.1), *Brassica napus* (ACA50526.1), *Lolium perenne* (AAY42598.1), *Arabidopsis thaliana* (AF256224.1), *Physcomitrella patens* (CAM96566.1) and *Thellungiella halophila* (BAJ34642.1).

The cDNA fragment spanning the entire open reading frame of *SbSOS1* was cloned and sequenced (GenBank accession number: EU879059). The *SbSOS1* cDNA was 3,774 bp long, contained a 90 bp 5^′^ UTR, a 3,480 bp open reading frame and a 204 bp 3^′^ UTR region. The cDNA encoded a polypeptide of 1,159 amino acid residues (Additional file 1: Figure S1) with a molecular mass of 128.4 kDa and an isoelectric point of 6.24. Hydrophobicity plot analysis predicted the presence of 11 strong transmembrane helices, beginning with the N-terminus outside and continuing till 447 residues (Additional file 1: Figure S1). The remaining C-terminal amino acid residues included a hydrophilic region and were predicted to reside in the cytoplasm. The SbSOS1 protein sequences were compared with the conserved domain in NCBI database and two conserved regions were identified: Nhap, an Na^+^/H^+^ antiporter spanning the transmembrane domain (amino acids 42 to 447) and a cyclic nucleotide-binding domain (cNMP; amino acids 753 to 857) that is located at the C-terminal tail (Figure 1a). The PSIPRED protein structure prediction server was used to predict the secondary structure, which included 55 coils, 45 alpha-helices and 18 beta-strands (Additional file 1: Figure S3). Amino acid alignments of the SbSOS1 sequence revealed a high degree of similarity to *Suaeda japonica* SjSOS1 (90%), *Chenopodium quinoa* CqSOS1 (83%)*, Mesembryanthemum crystallinum* McSOS1 (75%), *Populus euphratica* PeSOS1 (68%)*, Solanum lycopersicum* SlSOS1 (66%)*, Oryza sativa* OsSOS1 (62%)*, Thellungiella halophila* ThSOS1 (61%)*, Brassica napus* BnSOS1 (61%)*, Triticum aestivum* TaSOS1 (60%) and *Arabidopsis thaliana* AtSOS1 (57%) (Additional file 1: Figure S2). Phylogenetic relationships between these genes were examined by comparing the SbSOS1 protein to 21 other species (Figure 1b), which revealed that SbSOS1 was in close proximity to other halophytes, *Suaeda japonica* and *Chenopodium quinoa* at 100 bootstrap values resolving *Mesembryanthemum crystallinum* at another branch of 99 bootstrap values. Similarly, other members of the cruciferae and poaceae families resolve their members in the same cluster, which is indicative of evolutionary conservation among the members of different families.

### Differential expression analysis of *SbSOS1* as a result of exposure to salt stress

**Figure 2:**
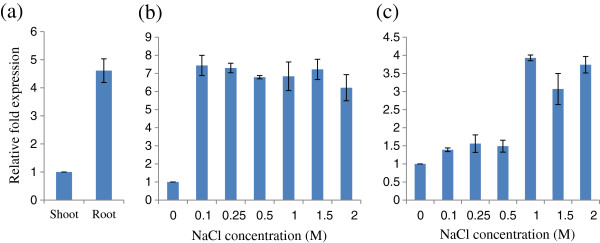
**Real**-**time PCR analysis of the *SbSOS1 *gene in response to NaCl stress.** (**a**) The relative expression of the *SbSOS1* transcript in root tissue compared with shoot tissue at 0 mM NaCl, (**b**) the level of *SbSOS1* transcript in root tissue at different NaCl concentrations (M), (**c**) *SbSOS1* transcript expression in shoot tissue at different NaCl concentrations (M). The relative expression in root tissue (**a**) was calculated using the C_T_ value of shoot tissue; in figures **b** and **c**, it was calculated using the values for 0 mM salt.

To study the expression of *SbSOS1,* real-time PCR was performed with cDNA from shoot and root tissues. Constitutively expressed *SbSOS1* was 4.5 times higher in root tissue than in shoot tissue (Figure 2a). In root tissue, its expression was increased by 7-fold at 100 mM NaCl, relative to 0 mM NaCl; however, *SbSOS1* expression remained constant at concentrations above 100 mM that were as high as 2.0 M (Figure 2b). In shoot tissue, *SbSOS1* expression increased from 1.5- to 4-fold in response to increasing NaCl concentration (Figure 2c).

### Overexpression of *SbSOS1* increases salinity tolerance

#### Analysis of T_0_ transgenic lines

**Figure 3:**
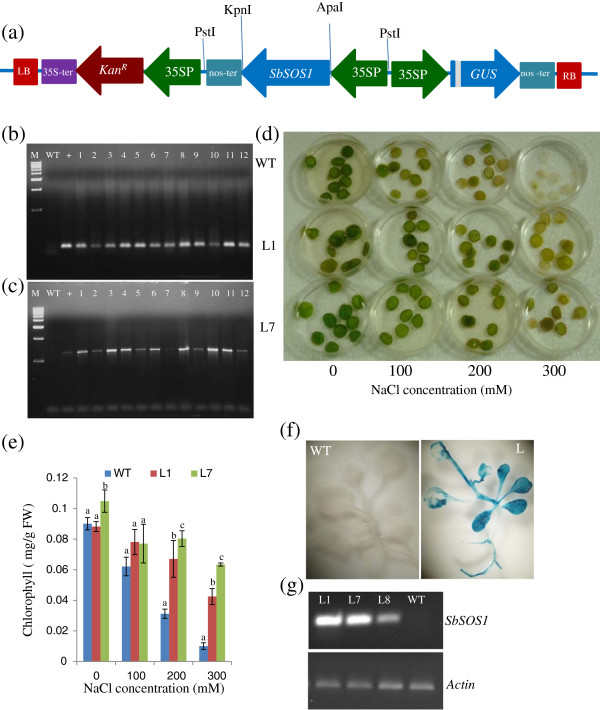
**Characterisation of transgenic tobacco plants.** (**a**) Schematic representation of the pCAMBIA2301 - 35S: *SbSOS1* construct used to transform tobacco plants with the *SbSOS1* gene. (**b**, **c**) Verification of transgenic lines via PCR analysis of the *SbSOS1* gene with real-time PCR primers and *gus*-specific primers in T_0_ plants. (**d**) Leaf disc assay of WT and transgenic lines (L1 and L7) at different NaCl concentrations in T_0_ plants. (**e**) The graph represents the mean and standard deviation (SD) of chlorophyll content in leaf discs of WT and transgenic lines (L1 and L7) at different NaCl concentrations. (**f**) GUS assay of T_1_ plants showing positive GUS expression in the transgenic line and negative expression in WT plants. (**g**) Transcript levels of the *SbSOS1* gene in transgenic lines (L1, L7 and L8) and WT T_1_ plants via semiquantitative RT-PCR.

The pCAMBIA2301-35S:SbSOS1 construct (Figure 3a) was introduced into tobacco plants for functional validation of *SbSOS1* gene. Putative transgenic lines were selected on kanamycin-containing medium and were subsequently verified by GUS analysis (Figure 3f). Seventy individual transgenic lines derived from independent transgenic events were analysed by the GUS assay. GUS-positive plants were subsequently transferred to plastic pots containing garden soil and again to earthen pots after 15 days of hardening.

The GUS-positive transgenic lines were further verified by PCR with gene specific real-time PCR primers (RT R and RT F) and *gus*-specific primers (gusA F and gusA R) (Figure 3b,c). Leaf disc assays were performed to determine the salinity tolerance of T_0_ transgenic plants. Leaf discs of uniform size from WT and transgenic plants (L1 and L7) were incubated in 0, 100, 200 and 300 mM NaCl. Leaf discs from WT plants began to turn yellow after 9 days and completely bleached after 17 days; in contrast, leaf discs from L1 and L7 plants remained relatively green (Figure 3d). To measure chlorophyll content in WT and transgenic plants, quantitative analyses were performed. The chlorophyll content in WT plants was significantly reduced as a function of increasing salt concentration. Of the two transgenic lines, L7 contained a larger amount of chlorophyll in the NaCl treatment and its chlorophyll content was not significantly affected in 200 and 300 mM NaCl relative to WT plants (Figure 3e).

#### Analysis of T_1_ transgenic lines

**Figure 4:**
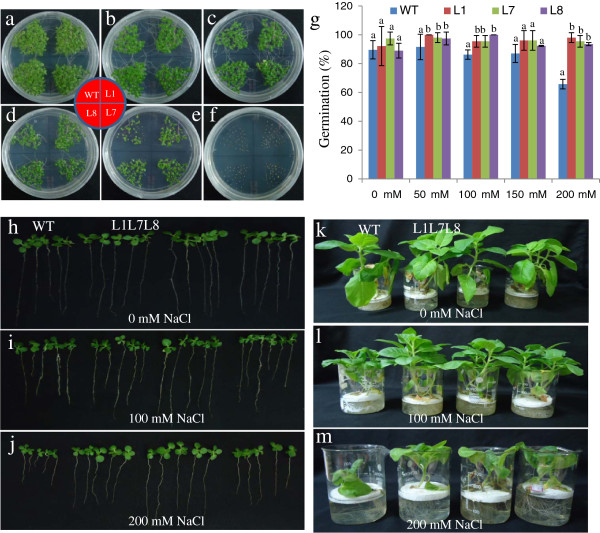
**Phenotypic comparison of the growth of WT and T1 transgenic lines** (**L1**, **L7 and L8**) **overexpressing the *SbSOS1 *gene.** (**a**-**f**) Germination of seeds from transgenic lines (L1, L7 and L8) and WT plants in (**a**) 0 mM, (**b**) 50 mM, (**c**) 100 mM, (**d**) 150 mM, (**e**) 200 mM, and (**f**) 300 mM NaCl. (**g**) The graph represents the per cent germination of transgenic lines (L1, L7 and L8) and WT plants in 0 mM, 50 mM, 100 mM, 150 mM, and 200 mM NaCl after 15 days. (**h**-**j**) Growth comparison of transgenic lines (L1, L7 and L8) and WT T_1_ seedlings in (**h**) 0 mM, (**i**) 100 mM, and (**j**) 200 mM NaCl. (**k**-**m**) Growth of whole plants from transgenic lines (L1, L7 and L8) and WT plants at different NaCl concentrations: (**k**) 0 mM, (**l**) 100 mM and (**m**) 200 mM in hydroponic culture.

**Figure 5:**
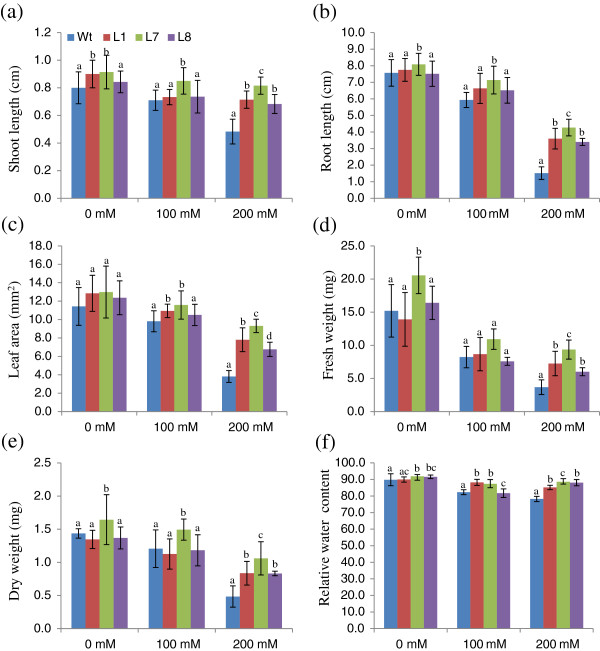
**Comparison of growth parameters of 30**-**day**-**old seedlings from transgenic lines** (**L1**, **L7 and L8**) **and WT plants in 0 mM**, **100 mM and 200 mM NaCl.** (**a**) shoot length, (**b**) root length, (**c**) leaf area, (**d**) fresh weight, (**e**) dry weight and (**f**) relative water content (RWC).

T_0_ seeds exhibited the expected 3:1 ratio of Kan^r^/ Kan^s^ during germination in kanamycin-containing medium. Three independent T_1_ transgenic lines were selected on the basis of GUS intensity and were further analysed for transgene expression via semi-quantitative RT-PCR (Figure 3g). To study the effect of salt stress on germination, WT and T_0_ seeds (L1, L7 and L8) were germinated in MS medium supplemented with 0, 50, 100, 150, 200 or 300 mM NaCl. Upon exposure to higher salt stress, the transgenic seeds exhibited better germination efficiency than WT seeds (Figure 4g). The efficiency of germination was reduced by increasing NaCl concentration for both WT and transgenic seeds (Figure 4a-g). In 300 mM NaCl, neither of the transgenic lines, nor the WT seeds, germinated until 15 days of seed inoculation (Figure 4f). In addition to seed germination assays, the growth of T_1_ transgenic seedlings exposed to salt stress conditions were also examined (Figure 4h-j). T_0_ seeds of WT and transgenic tobacco were allowed to germinate in MS medium for 8 days. Subsequently, T_1_ seedlings were transferred to medium containing 0, 100 or 200 mM NaCl (Figure 4h-j). The T_1_ transgenic lines exhibited significant enhancements in shoot and root length and leaf area relative to WT plants (Figure 5a-c). The transgenic lines also exhibited significant increases in both fresh and dry weight relative to WT (Figure 5d-e). The L7 transgenic line performed better in response to salt stress in terms leaf area, shoot and root length, relative water content and fresh and dry weight relative to both WT and other transgenic lines (Figure 4h-j, Figure 5a-f). The WT leaves exhibited dehydration in the presence of NaCl, whereas the transgenic lines were better hydrated and possessed higher relative water content (Figure 4h-j). Transgenic lines also exhibited better growth than their WT counterparts when subjected to salt stress in hydroponic culture (Figure 4k-m).

#### Electrolyte leakage, membrane stability and MDA content analysis

**Figure 6:**
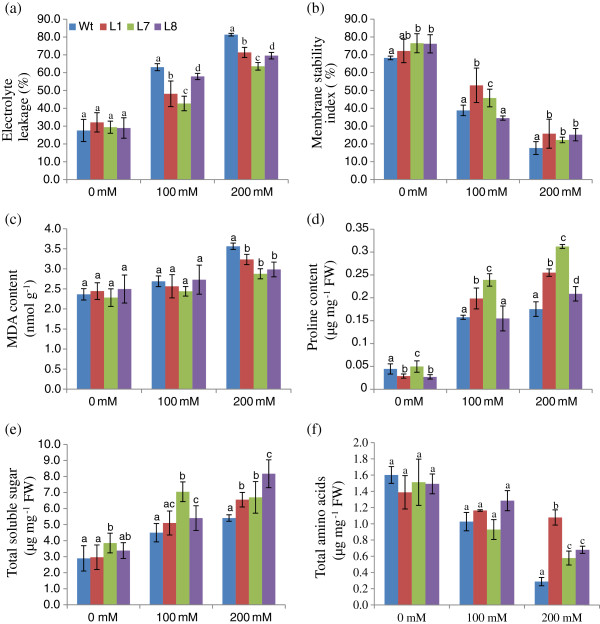
**Electrolyte leakage** (**a**), **membrane stability index** (**b**), **MDA** (**c**), **proline** (**d**), **total soluble sugar** (**e**), **and amino acid** (**d**) **contents of transgenic lines** (**L1**, **L7 and L8**) **and WT plants grown in hydroponic culture with 0 mM**, **100 mM**, **and 200 mM NaCl.**

Relative to WT plants, the transgenic lines exhibited significantly reduced electrolyte leakage during salt stress (Figure 6a). MSI analyses revealed that the cell membrane of lines L1 and L7 was more stable than WT plants (Figure 6b). Transgenic lines accumulated less MDA than WT in response to salt stress, which was indicative of reduced oxidative damage in plants overexpressing *SbSOS1* (Figure 6c).

#### Proline, total soluble sugar and amino acid content analysis

Proline, total soluble sugar and amino acid contents were measured in WT and transgenic plants to substantiate the function of the *SbSOS1* gene. At 0 mM NaCl, proline content was nearly identical in WT and transgenic plants; however, in the presence of 100 and 200 mM NaCl, the transgenic plants had higher proline content relative to WT (Figure 6d). Transgenic lines and WT both exhibited a gradual accumulation of total soluble sugar by increasing NaCl concentration; however, transgenic lines exhibited accumulation in all treatments that was greater than that of WT (Figure 6e). Whereas the total amino acid content decreased significantly in response to increased NaCl concentration for both WT and transgenic lines, the transgenic lines exhibited a more modest decrease in amino acid content relative to WT treated with 200 mM NaCl (Figure 6f).

#### *In vivo* localisation and quantification of O_2_^−^ and H_2_O_2_ in T_1_ plant leaves

**Figure 7:**
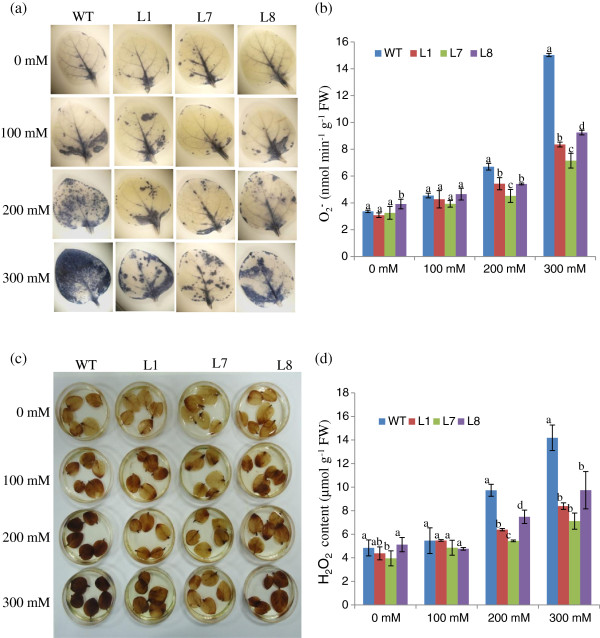
***In vivo *localisation and quantification of O**_**2**_^− ^**and H**_**2**_**O**_**2 **_**in leaves of *35S****-****SbSOS1 *and WT plants.** (**a**) Localisation of O_2_^−^ by NBT staining, (**b**) Quantification of O_2_^−^ content, (**c**) Localisation of H_2_O_2_ by DAB staining and (**d**) Quantification of H_2_O_2_ content.

Control, non-stressed leaves from WT and transgenic plants exhibited similar staining. In contrast, WT leaves exhibited more staining than transgenic lines in the presence of salt (Figure 7a,c). This result demonstrates that WT leaves accumulated more O_2_^−^ and H_2_O_2_ than transgenic lines, confirming that *SbSOS1* helps to minimise NaCl-induced oxidative stress *in situ*. Further these results were confirmed by quantification of O_2_^−^ and H_2_O_2_ accumulation (Figure 7b, d). Both O_2_^−^ and H_2_O_2_ contents were found higher in the WT plants compared to transgenic lines (Figure 7b,d).

#### Ion content analysis

##### Na^+^ content

**Figure 8:**
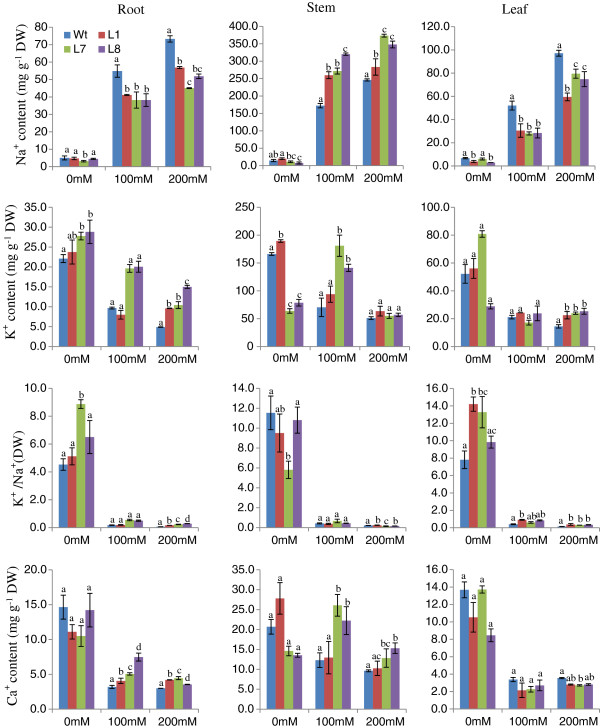
**Na**^+^, **K**^+ ^**and Ca**^**2**+ ^**contents in root**, **shoot and leaf tissues of transgenic lines** (**L1**, **L7 and L8**) **and WT plants grown in hydroponic culture with 0 mM**, **100 mM**, **and 200 mM NaCl.** Individual K^+^/Na^+^ ratios of different plant organs are also shown in the graphs.

**Figure 9:**
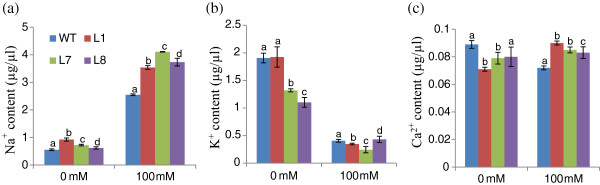
**Na**^+^, **K**^+ ^**and Ca**^**2**+ ^**contents in xylem sap of transgenic lines** (**L1**, **L7 and L8**) **and WT plants grown in hydroponic culture with 0 and 100 mM NaCl.**

At 0 mM NaCl (i.e., normal conditions) transgenic and WT plants exhibited approximately equal Na^+^ content in root, stem and leaf tissues individually. However, in the presence of 100 and 200 mM NaCl; root and leaf tissues exhibited significant reductions in Na^+^ ion content in all three transgenic lines (L1, L7 and L8) relative to WT plants (Figure 8). However, the accumulation of Na^+^ ions was found to be significantly higher in stem tissues in transgenic lines compared with WT. In general, stem tissues exhibited a 5-fold increase in Na^+^ content compared with root and leaf tissues. All transgenic lines exhibited higher concentrations of Na^+^ than WT in xylem sap under both normal and stress conditions (Figure 9).

##### K^+^ ion content

K^+^ content decreased as a function of increasing NaCl concentration in all tissues in both transgenic and WT plants (Figure 8). However, transgenic lines contained higher K^+^ content than WT in root, stem and leaf tissues. The maximum accumulation observed in stem tissue was 6-fold greater than that in leaf and root tissues. Transgenic lines and WT plants accumulated equivalent levels of K^+^ in xylem sap at 100 mM NaCl (Figure 9). Relative to WT, transgenic lines showed an increased K^+^/Na^+^ ratio in root and leaf tissues at all NaCl concentrations. In stem tissues, the transgenic lines and WT exhibited ratios that were nearly equal.

##### Ca^2+^ ion content

The transgenic lines showed greater Ca^2+^ content than WT in both root and stem tissues at both NaCl concentrations (Figure 8). Stem tissues exhibited a 2-fold increase in Ca^2+^ relative to root and leaf tissues. However, transgenic lines showed a slightly reduced Ca^2+^ content in leaf tissue relative to WT in stress conditions (Figure 8). Transgenic lines possessed a higher Ca^2+^ content in xylem sap compared with WT under stress conditions (Figure 9).

## Discussion

Salinity impedes plant growth and survival. Plants survive various stresses via mechanisms that operate on the cellular and molecular level. Salt stress can potentially perturb ion equilibrium in the plant system, and antiporters play an important role in adjusting cation concentrations to maintain cellular homeostasis. To avoid the toxic effects of salt, Na^+^ ions are compartmentalised into vacuoles, or extruded to the apoplast, and remain at low concentrations in the cytosol [50-53]. In response to salt stress, plants maintain high concentrations of K^+^ and low concentrations of Na^+^ in the cytosol by regulating the expression and activity of K^+^ and Na^+^ transporters and H^+^ pumps [54]. Until now, few reports have described the *in planta* overexpression of the *SOS1* gene from *Arabidopsis*[21,29]; *Thellungiella*[30] and *Puccinellia tenuiflora*[31]. Subsequently, a small number of reports have described the *SOS1* genes from different plant sources, which were characterised primarily in yeast systems [25,26,28,55,56]. To elucidate its function in salt tolerance, we have cloned the *SbSOS1* gene from the halophyte *Salicornia brachiata*. The SbSOS1 protein is predicted to contain 11 strong transmembrane regions (TM) at the amino-terminus, and a large cytoplasmic region at the carboxyl terminus (Additional file 1: Figure S1). The N-terminal portion that forms the membrane pore exhibited the greatest sequence homology, whereas C-terminal domains were less similar (Additional file 1: Figure S2). The TM3, TM5, TM8, TM10 and TM11 regions are highly conserved (Additional file 1: Figure S2). Amino acid alignment of SbSOS1 revealed a high degree of similarity to *Suaeda japonica* SjSOS1, *Chenopodium quinoa* CqSOS1*, Mesembryanthemum crystallinum* McSOS1, *Populus euphratica* PeSOS1*, Solanum lycopersicum* SlSOS1*, Oryza sativa* OsSOS1*, Thellungiella halophila* ThSOS1*, Brassica napus* BnSOS1*, Triticum aestivum* TaSOS1 and *Arabidopsis thaliana* AtSOS1 by 90%, 83%, 75%, 68%, 66%, 62%, 61%, 61%, 60% and 57%, respectively. A phylogenetic analysis revealed that SbSOS1 is closely related to *Caryophyllales* (*M. crystallinum*, *S. japonica*, *C. quinoa* and *Limonium gmelinii* Kuntze) (Figure 1b).

Real-time PCR revealed that the expression of *SbSOS1* remained higher in root tissues than in shoot tissues (Figure 2a). Similarly, Kant et al. [57] observed a 7-fold increase in *ThSOS1* transcript expression in roots relative to shoots. In shoot tissue, the expression was slightly increased in response to 0.5 M NaCl and increased significantly at higher concentrations. In contrast, even at 100 mM, expression increased by 7-fold in the root and remained constant thereafter (Figure 2b,c). Maughan et al. [58] also observed that *SOS1* expression was high at low salt concentration in the root tissue of *C. quinoa*, which indicates that the *SOS1* gene is hyper-inducible in the roots of halophytic plants at even low salt concentrations. Similarly, the *SOS1* gene from other plants was expressed more highly in the root in response to salt stress [17,27]. *P. euphratica PeSOS1* mRNA remained consistently high in shoot tissue when treated with 200 mM for 0, 24 or 48 h [28].

Transgenic plants overexpressing *SbSOS1* exhibited increased salt tolerance and higher rates of seed germination. Furthermore, in response to salt stress, transgenic plants exhibited increases in root length, shoot length, leaf area, fresh weight, dry weight, relative water content (RWC) and chlorophyll content relative to WT (Figures 3d-e: 4a-j: 5a-f). NaCl also causes the dehydration stress in plants because of the physiological drought stress. Munns and Tester [59] have mentioned that the salinity stress first causes the rapid osmotic stress followed by slow Na^+^ stress. The rapid osmotic stress in plants consecutively follows rapid dehydration. Oh et al. [30] studied that *Thellungiella Thsos1*-RNAi lines were highly salt sensitive and faster Na^*+*^ accumulating and showed severe water loss in shoots under salt stress and slower removal of Na^*+*^ from the roots compared with the WT. In the present study, the water retention was found higher in transgenic lines, confirming that *SbSOS1* help in water retention during Na^*+*^ stress to prevent from physiological desiccation. The chlorophyll content was found higher in *Arabidopsis* transgenic plants than WT in the presence of salt stress. The increase in chlorophyll content indicates that *SOS1* transgenic plants have better light harvesting and photosynthetic capacities than WT during salt stress [21]. The morphological features of the transgenic plants were similar to those of WT plants under normal growth conditions.

In the present study, transgenic plants experiencing salt stress exhibited low electrolyte leakage and greater cell membrane stability (Figure 6a-b). This observation was corroborated by the reduction in MDA. Analysis of the *in vivo* localisation of ROS via NBT and DAB revealed that *SbSOS1*-overexpressing lines maintained less ROS in response to salt treatment, which indicates a reduced potential for oxidative damage resulting from salt stress. Katiyar-Agarwal et al. [60] has reported that SOS1 interacts with *RCD 1* (regulator of oxidative stress responses) via its predicted cytoplasmic tail to regulate the expression of ROS-scavenging genes. The relative abundance of proline, total soluble sugar and amino acids are important biochemical indicators of salinity in plants [61]. Proline protects the plants in response to salt stress by regulating the accumulation of usable nitrogen, which contributes to membrane stability and mitigates the disruptive effect of NaCl. Transgenic plants overexpressing *SbSOS1* accumulate higher proline than WT plants during salt stress (Figure 6d). Total soluble sugar and amino acids are known to accumulate in plants during salt stress. *SbSOS1* transgenic lines accumulated higher levels of total soluble sugar and amino acids relative to WT plants (Figure 6e-f). Soluble sugars help transgenic plants to survive salt stress by regulating osmoregulation and by acting as molecular chaperones that stabilise protein structure [62]. Similarly, the increased accumulation of amino acids suggests a higher rate of protein synthesis in plants experiencing salt stress.

Studying ion partitioning in different plant tissues is important to fully understand the function of the transporter genes in that system. Previously, Shi et al. [11] observed that, at 100 mM NaCl, *sos1* mutant plants accumulated more Na^+^ in the shoot and xylem sap than WT, which suggests that *SOS1* functions in retrieving Na^+^ from the xylem stream in response to severe salt stress. Additionally, they overexpressed *AtSOS1* in *Arabidopsis*, which led to a reduced accumulation of Na^+^ in the shoot and xylem sap at 100 mM NaCl [21]. However, both of these studies were performed in *Arabidopsis* plants. As described by Olias et al. [27], *Arabidopsis* plants limit the precise dissection of the relative content of Na^+^ in stem vs. leaf; however, the ability to make this distinction is critical in assessing the role of SOS1 in xylem loading/unloading, as well as Na^+^ export in roots and retention in stems. Therefore, the present study was performed to examine the ion partition in different plant organs by overexpressing the *SbSOS1* gene in tobacco, which has more convenient anatomy [29]. Transgenic tobacco plants accumulated lower Na^+^ content in root and leaf at higher salt stress; in contrast, Na^+^ content was higher in stem and xylem sap relative to WT, which suggests a role in Na^+^ loading to xylem from root and leaf tissue. Whereas, Shi et al. [11] suggested that SOS1 function in retrieving Na^+^ from the xylem stream under severe salt stress, our study supports the hypothesis of Olias et al. [27], which suggests that the SOS1 functions in Na^+^ loading to xylem from root and leaf tissues.

Potassium is important to the plant system because it plays a role during plant growth and development. In response to NaCl stress, the *SbSOS1* transgenic lines showed higher K^+^ content in root and leaf tissue compared to WT plants. Transgenic lines showed higher ratios of K^+^/Na^+^ relative to WT plants in root and leaf tissues at all NaCl concentrations. Even under normal conditions, the transgenic lines exhibited high K^+^/Na^+^ ratios. However, in stem tissues, there was no significant difference between transgenic lines and WT plants (Figure 8). Wu et al. [13] showed that *sos1* mutants are hypersensitive to Na^+^ stress and defective in high-affinity K^+^ uptake; furthermore, these two phenotypes always co-segregated. Shi et al. [11] and Quintero et al. [24] demonstrated that a yeast strain (AXT3K) expressing *AtSOS1* accumulated less Na^+^ and exhibited improved K^+^ status compared with empty vector controls. It has also been reported that *AtSOS1* does not suppress defects of the yeast K^+^ transport mutant (*trk1 trk2)*[11,24,25] and aids indirect uptake and transport of K^+^ ions. Accordingly, *sos1* mutant plants displaced defective K^+^ uptake at low external concentrations, and their transport capacity was not altered by overexpression of the SOS1 protein at reduced K^+^ concentrations. However, *Cymodocea* SOS1 promoted uptake of K^+^ in bacteria [25]. Similarly, Wu et al. [28] revealed that *PeSOS1*-expressing bacteria maintained lower Na^+^ and higher K^+^ in the presence of 200 mM NaCl relative to vector alone, which resulted in an increase in the K^+^/Na^+^ ratio.

Stress and other extracellular stimuli influence intracellular Ca^2+^ concentration [63,64]. Ca^2+^-mediated signal transduction is considered one of the earliest events in salt signalling and plays an essential role in ion homeostasis that permits salt tolerance in plants [54]. The *SbSOS1* transgenic lines showed higher Ca^2+^ content than WT in root and stem tissues at both NaCl concentrations (Figure 8). However, transgenic lines showed slightly lower Ca^2+^ content in leaf tissues relative to WT plants exposed to salt stress (Figure 8). Transgenic lines exhibited increases in Ca^2+^ content relative to WT plants in xylem sap at stress conditions (Figure 9). Similar to our studies, Guo et al. [65] proposed a link between Ca^2+^ transport and SOS1 activity, and also demonstrated that the *sos* mutant alters the activity of Ca^2+^ transport systems in both normal and NaCl stress conditions. In WT plants, the Ca^2+^ influx rate increased after a 5 min treatment with NaCl, resulting in an increase in cytoplasmic Ca^2+^. During the same time interval, Ca^2+^ efflux was observed in *sos1* mutants, which resulted in a decrease in cytoplasmic Ca^2+^. The variability of Ca^2+^ content in the *SbSOS1* transgenic lines, compared with WT, can be attributed to changes in Ca^2+^ transporter activity. Therefore, it is possible that SbSOS1 affects the Ca^2+^ transporter.

## Conclusions

In conclusion, the *SbSOS1* gene from the extreme halophyte *S. brachiata* was cloned which showed expression during salt stress*. SbSOS1* expression was consistently higher in the root tissue than shoot tissue and was upregulated by salt stress. Overexpression of *SbSOS1* in tobacco conferred a high degree of salt tolerance, enhanced plant growth and altered physiological and biochemical parameters in response to salt stress. Furthermore, the transgenic lines exhibited lower levels of MDA and ROS accumulation as a result of reduced cytosolic Na^+^ content and oxidative damage. Transgenic tobacco plants accumulated reduced Na^+^ content in root and leaf tissues, but higher Na^+^ content in stem and xylem sap, relative to WT, which resulted from enhanced Na^+^ loading in xylem from root and leaf tissues. This finding demonstrates that, in addition to the Na^+^ efflux outside the plasma membrane, SbSOS1 transporter also helps to maintain differing concentrations of Na^+^ in various organs. These results support a broader role of *SbSOS1 in planta* and suggest that this gene can be utilised to develop salt-tolerant transgenic crops in the future.

## Authors’ contributions

NSY has carried out gene cloning, raised transgenic plants and analyzed them for functional validation. PSS and AJ carried out the biochemical assay of transgenic lines. PKA and BJ designed, coordinated the experiments and finalized MS. All authors read and approved the final manuscript.

## Supplementary Material

Additional file 1 **Figure S1.** Nucleotide sequence and deduced amino acid sequence of *SbSOS1.* The amino acid residues are indicated by a single letter code. The 11 putative transmembrane domains (TM) are underlined. **Figure S2.** Comparison of amino acid alignment of SbSOS1 (ACJ63441) with Na^+^/H^+^ antiporters SjSOS1 from *Sueda japonica* (BAE95196), CqSOS1 from *Chenopodium quinoa* (ACN66494), ThSOS1 from *Thellungiella halophila* (ABN04857), SlSOS1 from *Solanum lycopersicum* (CAG30524), AtSOS1 from *Arabidopsis thaliana* (AAD20091), PeSOS1 from *Populus euphratica* (ABF60872) and OsSOS1 from *Oryza sativa* (AAW33875). Identical amino acids are highlighted in black, while conservative substitutions are highlighted in pink. **Figure S3.** Secondary structure of SbSOS1 protein. Helix, strands and coils are indicated by green rods, arrow and solid lines. Click here for file
