# Supplementary figures and images for: The SbSOS1 gene from the extreme halophyte Salicornia brachiata enhances Na+ loading in xylem and confers salt tolerance in transgenic tobacco
Source: BMC Plant Biol. 2012 Oct 11;12:188. doi: 10.1186/1471-2229-12-188 (PMC3548769; doi:10.1186/1471-2229-12-188)

## Slide 1
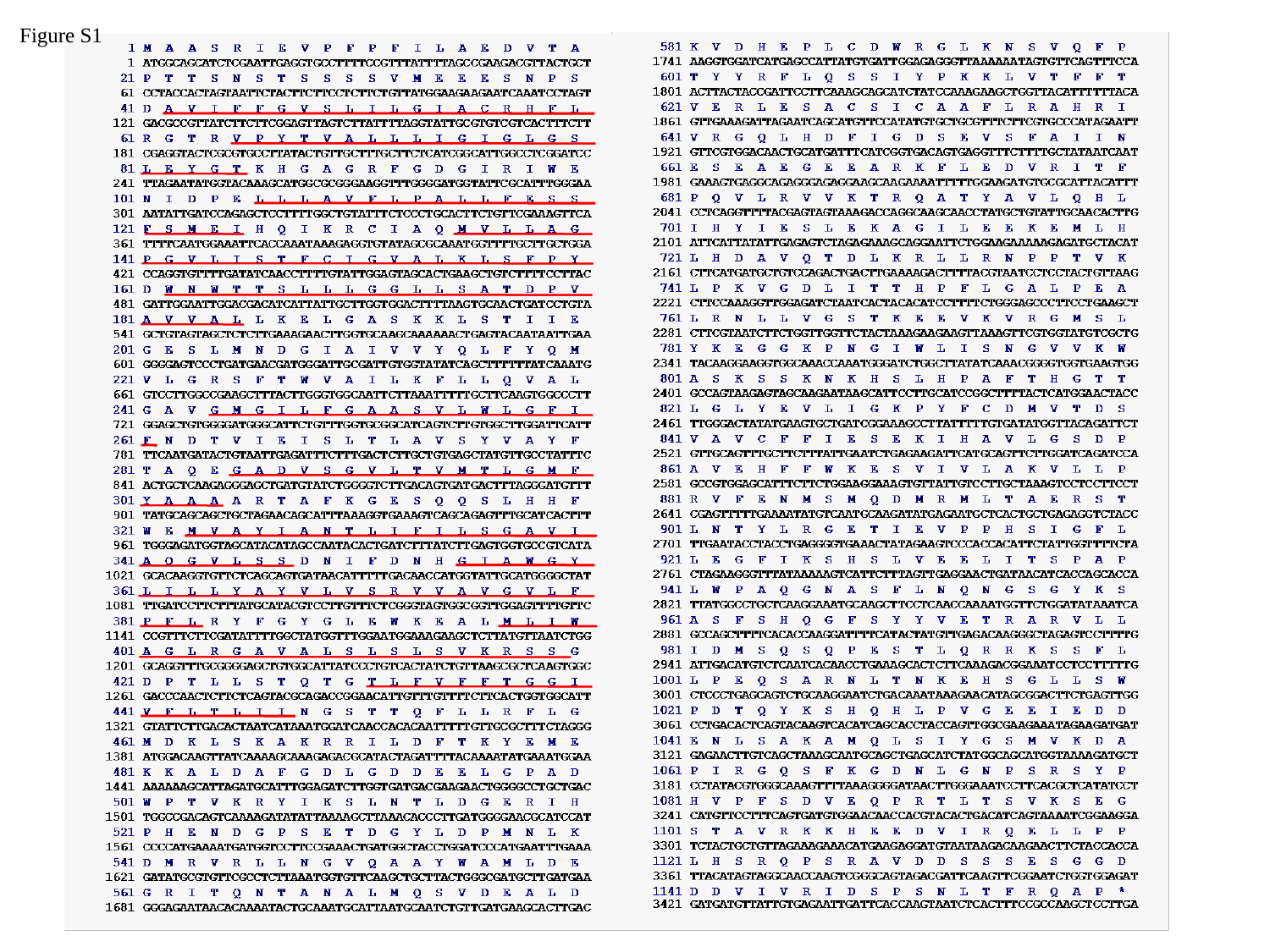

Figure S1

## Slide 2
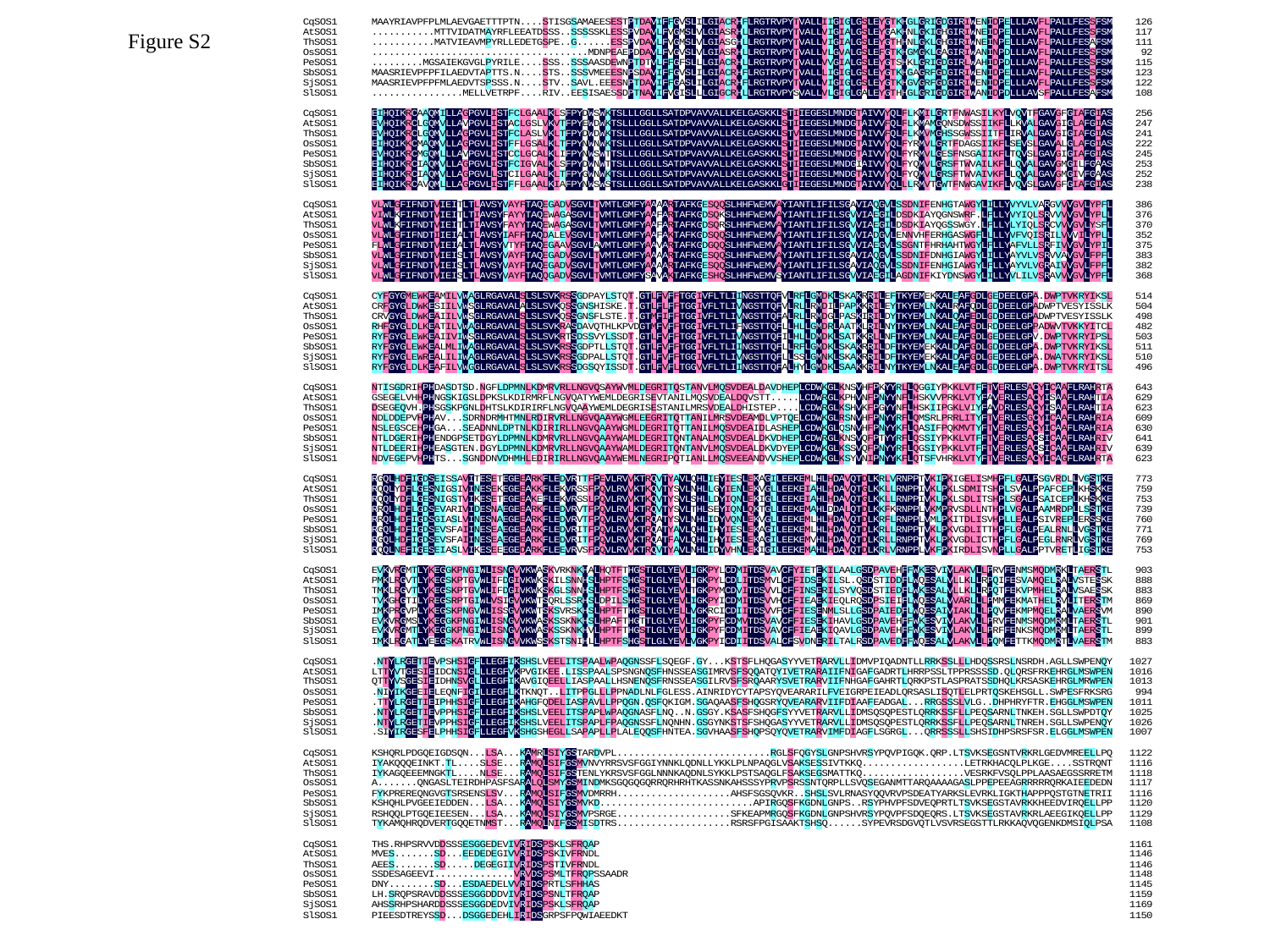

Figure S2

## Slide 3
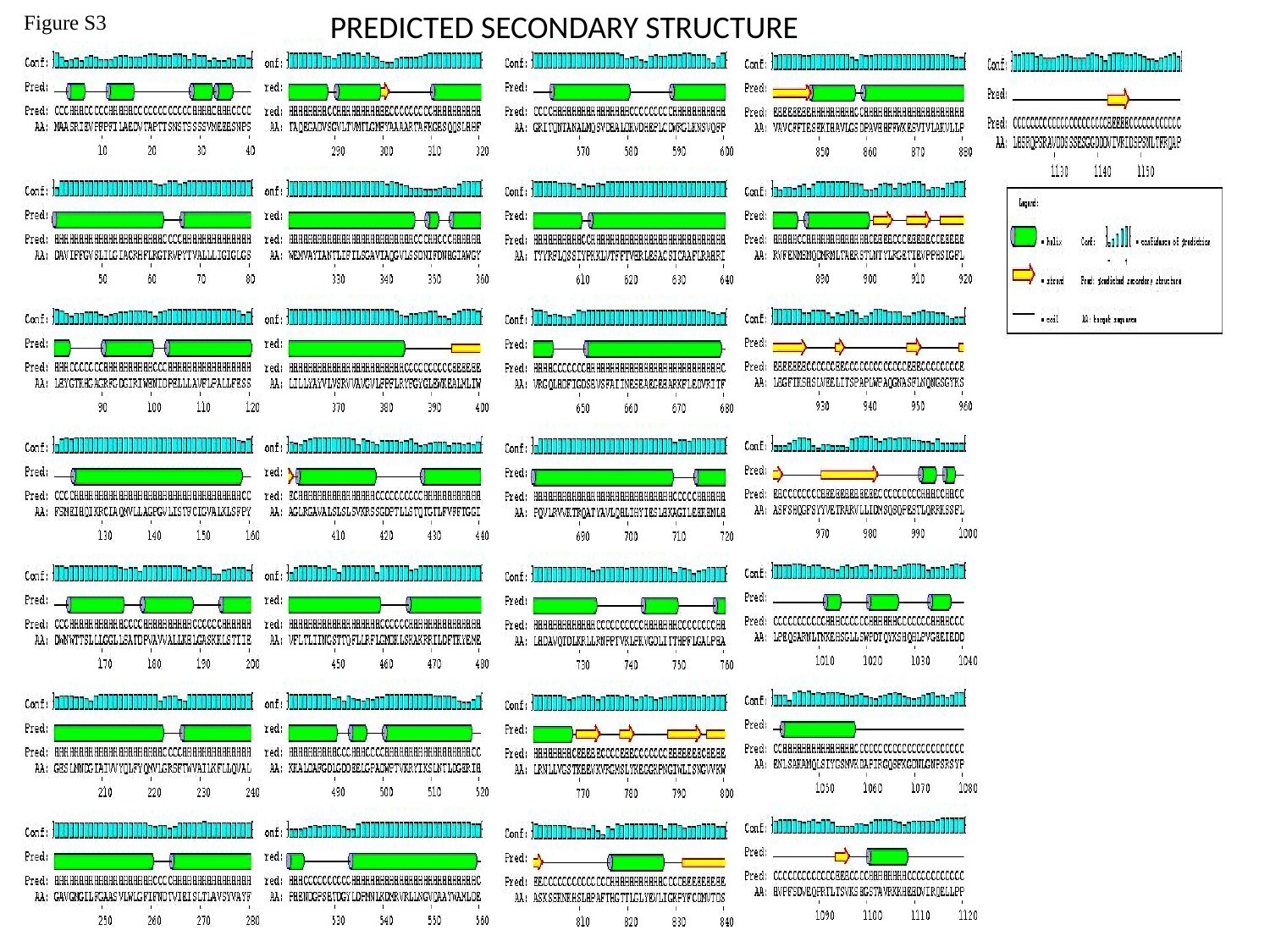

PREDICTED SECONDARY STRUCTURE
Figure S3

Supplement: Additional file 1 — Figure S1. Nucleotide sequence and deduced amino acid sequence of SbSOS1. The amino acid residues are indicated by a single letter code. The 11 putative transmembrane domains (TM) are underlined. Figure S2. Comparison of amino acid alignment of SbSOS1 (ACJ63441) with Na+/H+ antiporters SjSOS1 from Sueda japonica (BAE95196), CqSOS1 from Chenopodium quinoa (ACN66494), ThSOS1 from Thellungiella halophila (ABN04857), SlSOS1 from Solanum lycopersicum (CAG30524), AtSOS1 from Arabidopsis thaliana (AAD20091), PeSOS1 from Populus euphratica (ABF60872) and OsSOS1 from Oryza sativa (AAW33875). Identical amino acids are highlighted in black, while conservative substitutions are highlighted in pink. Figure S3. Secondary structure of SbSOS1 protein. Helix, strands and coils are indicated by green rods, arrow and solid lines. [file 1471-2229-12-188-S1.ppt]
